# Ambient PM gross β-activity and glucose levels during pregnancy

**DOI:** 10.1186/s12940-021-00744-9

**Published:** 2021-06-14

**Authors:** Veronica A. Wang, Tamarra James-Todd, Michele R. Hacker, Karen E. O’Brien, Blair J. Wylie, Russ Hauser, Paige L. Williams, Andrea Bellavia, Marlee Quinn, Thomas F. McElrath, Stefania Papatheodorou

**Affiliations:** 1 Department of Environmental Health, Harvard T.H. Chan School of Public Health, Boston, MA 02115 USA; 2 Department of Epidemiology, Harvard T.H. Chan School of Public Health, Boston, MA 02115 USA; 3 Department of Obstetrics and Gynecology, Beth Israel Deaconess Medical Center, Harvard Medical School, Boston, MA 02115 USA; 4 Department of Biostatistics, Harvard T.H. Chan School of Public Health, Boston, MA 02115 USA; 5 Division of Maternal-Fetal Medicine, Brigham & Women’s Hospital, Harvard Medical School, Boston, MA 02115 USA; 6 Harvard T.H. Chan School of Public Health, 677 Huntington Avenue, Kresge Bldg, Boston, MA 02115 USA

**Keywords:** Particle radioactivity, Blood glucose, Pregnancy, Glucose metabolism, Pregnancy complications, Gestational diabetes

## Abstract

**Background:**

Exposure to ionizing radiation has been associated with insulin resistance and type 2 diabetes. In light of recent work showing an association between ambient particulate matter (PM) gross β-activity and gestational diabetes mellitus (GDM) among pregnant women, we examined pregnancy glucose levels in relation to PM gross β-activity to better understand this pathway.

**Methods:**

Our study included 103 participants receiving prenatal care at Beth Israel Deaconess Medical Center in Boston, MA. PM gross β-activity was obtained from US Environmental Protection Agency’s RadNet program monitors, and blood glucose levels were obtained from the non-fasting glucose challenge test performed clinically as the first step of the 2-step GDM screening test. For each exposure window we examined (i.e., moving average same-day, one-week, first-trimester, and second-trimester PM gross β-activity), we fitted generalized additive models and adjusted for clinical characteristics, socio-demographic factors, temporal variables, and PM with an aerodynamic diameter ≤ 2.5 μm (PM_2.5_). Subgroup analyses by maternal age and by body mass index were also conducted.

**Results:**

An interquartile range increase in average PM gross β-activity during the second trimester of pregnancy was associated with an increase of 17.5 (95% CI: 0.8, 34.3) mg/dL in glucose concentration. Associations were stronger among younger and overweight/obese participants. Our findings also suggest that the highest compared to the lowest quartile of one-week exposure was associated with 17.0 (95% CI: − 4.0, 38.0) mg/dL higher glucose levels. No associations of glucose were observed with PM gross β-activity during same-day and first-trimester exposure windows. PM_2.5_ was not associated with glucose levels during any exposure window in our data.

**Conclusions:**

Exposure to higher levels of ambient PM gross β-activity was associated with higher blood glucose levels in pregnant patients, with implications for how this novel environmental factor could impact pregnancy health.

**Supplementary Information:**

The online version contains supplementary material available at 10.1186/s12940-021-00744-9.

## Background

Gestational diabetes mellitus (GDM), glucose intolerance first identified during pregnancy, affects 3 to 14% of pregnancies in the US every year [1–3], and the prevalence continues to rise. GDM is associated with increased risk of several pregnancy complications, including cesarean delivery, preeclampsia, and neonatal hypoglycemia [4, 5]. Although most women with GDM return to normal glucose tolerance after delivery, up to approximately 50% of women with GDM develop type 2 diabetes within 5 to 10 years following delivery, with concomitant increased risk of cardiovascular disease [4, 6, 7]. In addition to an increased risk of type 2 diabetes, GDM confers an increased risk of long-term adverse outcomes for offspring after delivery, including obesity and carbohydrate intolerance that can lead to increases in metabolic syndrome and cardiac disease in adult life [8]. Interestingly, these adverse outcomes also are linked to elevated pregnancy glucose levels that do not meet the clinical threshold for gestational diabetes [9]. As such, identifying modifiable risk factors that reduce elevated pregnancy glucose levels is of great importance.

Several air pollutants, specifically nitrogen dioxide, nitrogen oxides, sulfur dioxide, and particulate matter (PM) with an aerodynamic diameter ≤ 2.5 μm (PM_2.5_), have been previously identified to be associated with an increased risk of GDM [10]. While PM_2.5_ during pre-pregnancy and the first trimester appear to have a null association with GDM, second-trimester exposure to PM_2.5_ has been found to be associated with glucose metabolism [10]. Oxidative stress and inflammation from PM_2.5_ are thought to be the primary mechanisms by which this exposure might increase GDM risk [11]. However, the properties that make PM toxic and can lead to the development of GDM have not been elucidated. A property that has been proposed is particle radioactivity, the radioactive component of PM in the air, which also is associated with markers of inflammation and oxidative stress [12].

As described by Porstendorfer [13] and others [14–16], freshly generated radon (Rn) progeny initially (within 1 s) form unattached respirable ultrafine clusters with diameters from 0.5 to 5 nm, and then (within 1–100 s) attach to larger particles in the ambient and indoor air to form particle radioactivity. When inhaled, the attached and unattached particulate Rn progeny deposit in the lungs, and can translocate to other organs, where they continue to decay and emit α-, β- and ɣ-radiation (internal radiation) [17]. External α- and most β-radiation cannot penetrate the intact epidermis; thus, exposure to α- and β-radiation can only occur via inhalation and ingestion of Rn and particle radioactivity. According to the US Environmental Protection Agency (EPA), 73% of a person’s natural background exposure to radioactivity is through inhalation, most of which is radioactive gases Rn and thoron from the natural breakdown of primordial radionuclides, uranium and thorium [18]. Rn gas, specifically the Rn-222 radioisotope, has a 3.8 day half-life and decays to α-, β-, and γ-radiation-emitting isotopes of elements such as polonium (218Po, 214Po, and 210Po) and lead (214Pb and 210Pb), among others, referred to as Rn progeny [17]. Gross β-activity has been previously shown to be well correlated with gross α-activity in airborne particulate samples (*R* = 0.72) [19] and can, therefore, be used as a surrogate for particle radioactivity. In a large cohort study of pregnant women in Massachusetts, PM gross β-activity was also used to represent particle radioactivity and was found to be associated with increased odds of GDM (OR: 1.18, 95% CI: 1.10, 1.22) [20]. However, the pathways through which exposure to radioactive particles in the air can lead to GDM remain uncertain. Furthermore, it remains unclear whether PM gross β-activity more subtly impacts pregnancy glucose levels without manifesting GDM, with pregnancy being a sensitive window of exposure for low-dose inhaled radiation, given the increased insulin-resistant state [21].

Therefore, we sought to investigate the association between PM gross β-activity and glucose levels in pregnancy. This association was evaluated by assessing four exposure time windows (same-day, one-week, first trimester, second trimester) as they related to pregnancy glucose levels from standard GDM screening. We also hypothesized that the association between PM gross β-activity and pregnancy glucose levels could vary by maternal age and body mass index (BMI), well-established risk factors for GDM.

## Methods

### Study population

Patients who received prenatal care in the Department of Obstetrics and Gynecology at Beth Israel Deaconess Medical Center were recruited before 15 weeks of gestation as part of the Environmental Reproductive and Glucose Outcomes Study (ERGO), which initiated recruitment in December 2016 [22]. Participants were at least 18 years old and did not have preexisting type 1 or type 2 diabetes at enrollment. Although ERGO initiated recruitment in December 2016, recruitment from the Beth Israel Deaconess Medical Center began in 2018. Our study includes participants who had completed the 50-g non-fasting glucose challenge test (GCT) at the time of analysis, which includes years 2018 and 2019 (*N* = 120). The study population was restricted to participants who underwent the GCT (*N* = 119) and had complete exposure and covariate data, resulting in a final sample size of 103 participants.

### PM gross β-activity (Bq/m^3^)

We used PM gross β-activity measured by EPA RadNet monitors. The EPA RadNet system was established to monitor background environmental radiation through routine analyses of precipitation, drinking water, and air filters across the US [23]. Each monitor collects total suspended particles (TSP) on a 4-in. diameter polyester fiber filter, and most of the measured TSP radioactivity is associated with PM_2.5_ [24]. This is because ultrafine radionuclides attach most readily to the PM accumulation mode (sizes between 0.1 and 1 μm). Therefore, even though RadNet measures are made on TSP samples, they adequately represent exposure to PM gross β-activity for health studies. Air filter samples were collected over several days (mean = 3.2 days) and were sent to the National Analytical Radiation Environmental Laboratory for analysis of gross β-activity concentration using a survey meter with beta channels or a Geiger-Muller counter with beta calibrated detector probes [25].

We assigned exposure to PM β-activity by linking the measurements from the closest RadNet monitor to the participant’s residential zip code on the date of the glucose test. There are 7 RadNet monitors in Greater Massachusetts: Portland, ME; Concord, NH; Boston, MA; Worcester, MA; Providence, RI; Hartford, CT; and Albany, NY. Based of the spatial distribution of the participants’ residential zip codes, we included measurements from the closest monitors located at Boston, MA and Worcester, MA. RadNet monitors are sometimes missing days of data. To impute missing days, a separate random forest model [26] was created for each monitor using beta values from nearby monitors, meteorological variables from the National Centers for Environmental Prediction’s North American Reanalysis, and information on air mass trajectories [27]. Models were fit using R package “h2o”, and model parameters were tuned by minimizing the out-of-bag mean square error, and results were validated using 10-fold cross-validation. The overall cross-validated R^2^ ranged from 0.80 to 0.86.

### Exposure windows

Because there is limited understanding of the relevant exposure window of PM gross β-activity for glucose concentration, we explored four exposure windows: moving averages of same-day, one-week, first-trimester, and second-trimester PM gross β-activity. The same-day exposure window is the average PM gross β-activity on the day of the GCT used to screen for GDM. The one-week exposure window was calculated by taking an average of the PM gross β-activity values for the day of the examination and the 6 daily values prior to the GCT. The first-trimester exposure window included exposure from the 1st through the 12th week of gestation [28], and the second-trimester exposure window included exposure from the 13th through the 28th week of gestation [28] or until the date of the GCT.

### Blood glucose levels (mg/dL)

We used blood glucose levels from the non-fasting, 50-g GCT performed at a median of 26.3 weeks of gestation (range: 23.3–30.3) in this sample. GCT is the first step in the 2-step screening for GDM, and the Carpenter-Coustan criteria [29] was used for diagnosis of GDM. Accordingly, if a woman had a blood glucose level ≥ 140 mg/dL for the GCT, she received further testing and took a 3-h, fasting 100-g oral glucose tolerance test. For purposes of the present analysis, we treated GCT values that all participants took as a part of their GDM screening to measure pregnancy glucose intolerance as a continuous variable [30].

### Covariates

Self-reported maternal covariates were collected through several short questionnaires prior to the GCT, including pre-pregnancy BMI in kg/m^2^, date of birth, race/ethnicity, educational attainment, and insurance status. The participant’s age in years was derived using the reported date of birth and the date of informed consent for participation in the study. Participants were asked to indicate all of the race/ethnicity categories that they self-identified with: White/Caucasian, Black/African American, Haitian/Caribbean, Native Hawaiian or other Pacific Islander, South Asian, East Asian, American Indian/Alaskan Native, Hispanic or Latino, Other, Please specify, and/or Refuse to answer. Participants also were asked to indicate their highest level of education (Less than 12th grade, Graduated from high school or GED, Some college/completed an associate’s degree, Graduated from college (4 years), or Graduate degree) and the type of insurance they had (Self pay, Private Insurance/ HMO, Medicaid/ SSI/ Mass Health, None, Unsure).

Zip code level covariates including, residential median neighborhood income in US dollars, median value of owner-occupied housing in US dollars, and percent open space, were derived from the 2010 Census. Percent open space was calculated by dividing the total square miles of open space by the total square miles of area in a zip code and then multiplying by 100. Daily temperature in Fahrenheit was obtained from the National Oceanic and Atmospheric Administration at Boston Logan International Airport [31] and converted to Celsius.

Although PM_2.5_ (μg/m^3^) is the vector of internal radioactivity, it is not necessarily indicative of the amount of radioactivity attached. PM_2.5_ estimates were obtained from the closest EPA monitors to participant residences, including those at Boston, Brockton, Chelmsford, Greenfield, Haverhill, Lynn, Pittsfield, Springfield, and Worcester in Massachusetts. We created the same exposure windows for PM_2.5_ (same-day, one-week, first-trimester and second-trimester).

### Statistical analysis

We used generalized additive models (GAMs) to evaluate the effect of PM gross β-activity on non-fasting GCT results and adjusted for covariates identified based on prior knowledge. Separate GAMs were fit to evaluate the effect of ambient PM gross β-activity on glucose levels for each of the four exposure windows (same-day, one-week, first-trimester, second-trimester). Spearman correlation coefficients were computed for first- and second- trimester PM_2.5_ with PM gross β-activity. In order to account for all distinct time-varying exposures simultaneously, we also included first- and second- trimester exposures in the same model as additional analyses.

In all multivariable models, we adjusted for individual-level covariates, including pre-pregnancy BMI (continuous), age (continuous) at enrollment, race/ethnicity (White versus non-White), educational attainment (college or less versus graduate school), and insurance status (private versus other) to improve precision. That is, these variables have been shown to be strong predictors of blood glucose levels during pregnancy in previous studies [32, 33]. Median neighborhood income (continuous), median value of owner-occupied housing (continuous) and percent open space (continuous) [34] were used to characterize neighborhood socioeconomic status and also were adjusted for. Furthermore, we included outdoor temperature (continuous) on the day of the glucose measurement. Relative humidity did not change the effect estimates of glucose (data not shown) and were therefore not included in the model. Using a penalized spline, the model included a variable representing date to take seasonality and time trend into account. We visually checked all other continuous variables for departure from linearity with the outcome using penalized splines, which was not the case for any variable (data not shown). The estimates were scaled to the interquartile range (IQR) (25th to 75th percentile) and reported with their 95% confidence intervals (CIs). Each estimate gives the change in glucose in mg/dL for each IQR increase in PM gross β-activity. The change in pregnancy glucose level across quartiles of PM gross β-activity exposure was also examined. We fit models with and without adjusting for PM_2.5_ in the corresponding exposure windows to estimate the independent effect of PM gross β-activity on glucose levels. Additional models were fit to evaluate the association between PM_2.5_ and glucose concentrations (without adjusting for PM gross β-activity) in our study population. As sensitivity analyses, we fit the model with insurance status imputed using multiple imputation by chained equations because it was the only covariate that had over 10% missingness (10.1%) along with the model with all missing covariates imputed as sensitivity analyses.

In light of previous evidence that the effect of air pollution on GDM is stronger in younger [35] and overweight/obese [36] participants, we conducted separate stratified analyses by the median age of 32 years in our sample and by BMI of 25 [37] for each exposure window. All statistical analyses were performed with R version 4.0.0 (R Development Core Team, Vienna, Austria).

## Results

Participants had a mean (standard deviation) age of 32.7 (4.6) years and BMI of 24.9 (4.7) kg/m^2^ (Table 1). The participants were well-educated; all had attained at least a high school diploma, and 53% had completed graduate school. Non-Hispanic white participants represented the largest racial/ethnic group (66%). Overall, we observed a mean (standard deviation, range) PM gross β-activity concentration and PM_2.5_ of 2.0 (0.3, 1.5–2.8) × 10^− 4^ Bq/m^3^ and 6.9 (1.6, 3.6–10.1) μg/m^3^, respectively. Socio-demographic characteristics in this study were not only similar across each PM gross β-activity quartile but also were similar to those of the overall ERGO cohort, as shown in Supplemental Table 1. PM_2.5_ and PM gross β-activity measurements were weakly correlated, and first and second trimester PM gross β-activity had a moderately negative correlation (Supplemental Table 2). This population had an average (standard deviation) glucose concentration of 104.6 (28.7) mg/dL. Eight participants had a glucose level above 140 mg/dL, one standard GCT clinical cut-off for additional GDM screening [38], and one participant was later diagnosed with GDM.

**Table 1:** Socio-demographic characteristics of the 103 participants by quartiles of PM gross β-activity

	First-trimester PM gross β-activity, 10^**− 4**^ Bq/m^**3**^				
	Total ***N*** = 103	Q1 (1.5–1.7) ***N*** = 25	Q2 (1.8–2.0) ***N*** = 26	Q3 (2.0–2.2) ***N*** = 29	Q4 (2.2–2.8) ***N*** = 23
**PM gross β-activity,** **10**^**− 4**^ **Bq/m**^**3**^	2.0 (0.3)	1.6 (0.1)	1.9 (0.1)	2.1 (0.1)	2.3 (0.2)
**PM**_**2.5**_**,** μg**/m**^**3**^	6.9 (1.6)	6.4 (1.1)	6.8 (1.8)	7.4 (1.6)	7.0 (1.8)
**Temperature, °C**	11.2 (9.8)	10.8 (7.9)	8.7 (8.5)	10.9 (9.9)	14.9 (12.2)
**Age, years**	32.7 (4.6)	33.0 (4.9)	32.2 (3.9)	33.5 (5.0)	32.0 (4.7)
**Body mass index, kg/m**^**2**^	24.9 (4.7)	24.5 (3.4)	25.1 (4.6)	24.7 (5.0)	25.3 (5.7)
**Highest educational attainment**					
High school/College	48 (46.6%)	10 (40.0%)	13 (50.0%)	14 (48.3%)	11 (47.8%)
Graduate school	55 (53.4%)	15 (60.0%)	13 (50.0%)	15 (51.7%)	12 (52.2%)
**Race/ethnicity**					
White	68 (66.0%)	15 (60.0%)	18 (69.2%)	18 (62.1%)	17 (73.9%)
Non-White	35 (34.0%)	10 (40.0%)	8 (30.8%)	11 (37.9%)	6 (26.1%)
**Prenatal insurance type**					
Private	88 (85.4%)	22 (88.0%)	22 (84.6%)	25 (86.2%)	19 (82.6%)
Other	15 (14.6%)	3 (12.0%)	4 (15.4%)	4 (13.8%)	4 (17.4%)
% Open space*	1.2 (1.4)	1.4 (1.8)	0.7 (0.7)	1.5 (1.9)	1.0 (0.7)
Median household income*, $K	54.1 (16.8)	54.5 (15.7)	59.1 (18.3)	46.8 (12.2)	57.2 (18.9)
Median value of owner-occupied housing*, $K	308 (192)	306 (191)	357 (230)	295 (187)	270 (147)

Values of continuous variables were reported as mean (standard deviation, range), while values of categorical variables were reported as n (%).

Q1 through Q4 = participants in first through fourth quartile of PM gross β-activity exposure level.

*in census tract

**Fig. 1:**
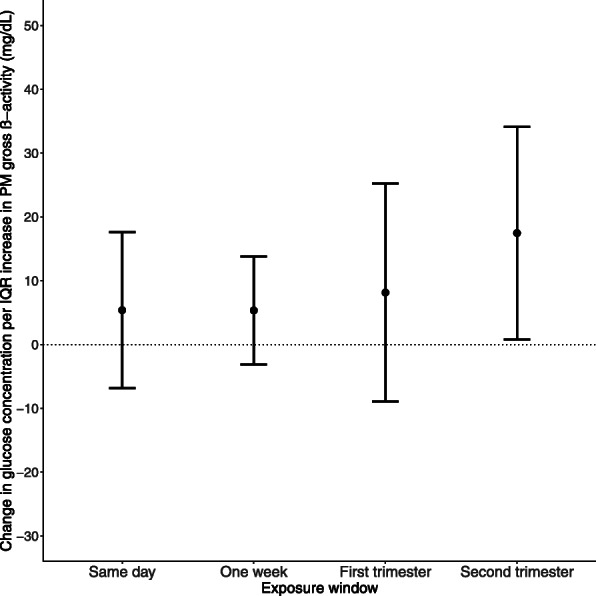
Changes in glucose concentration per interquartile range (IQR) increase in PM gross β-activity for each exposure window: the day of GCT, the week prior, the first trimester, and the second trimester. All models were adjusted for PM_2.5_, temperature, pre-pregnancy body mass index, maternal age, maternal race/ethnicity, maternal educational attainment, insurance status, median neighborhood income, median value of owner-occupied housing, and percent open space. The x-axis represents the exposure window, and the y-axis represents the change in glucose concentration in mg/dL per IQR increase in PM gross β-activity. The error bars denote the 95% confidence intervals

Figure 1 shows the change in glucose concentration per IQR increase in PM gross β-activity for the exposure windows examined. An IQR increase in average PM gross β-activity the day of the GCT, the week prior to the GCT, and during the first trimester of pregnancy were not significantly associated with glucose levels, 6.0 (95% CI: − 6.2, 18.2), 5.5 (95% CI: − 3.1, 14.0), and 4.4 (95% CI: − 4.5, 13.2) mg/dL, respectively. However, an IQR increase in average PM gross β-activity during the second trimester of pregnancy was found to be significantly associated with 17.5 (95% CI: 0.8, 34.3) mg/dL higher pregnancy glucose levels. In models without PM gross β-activity, PM_2.5_ was not associated with glucose levels in this population (Supplemental Fig. 1). The association between PM gross β-activity and GCT glucose level remained similar with and without adjusting for PM_2.5_ (Supplemental Fig. 2). When we adjusted simultaneously for first- and second-trimester exposures, the results were essentially the same (data not shown). The results were also consistent with the findings noted above when we used multiple imputation to account for missing information on insurance status (Supplemental Table 3). In addition, when we imputed values for all missing covariates, the results were attenuated but in the same direction (data not shown).

**Fig. 2:**
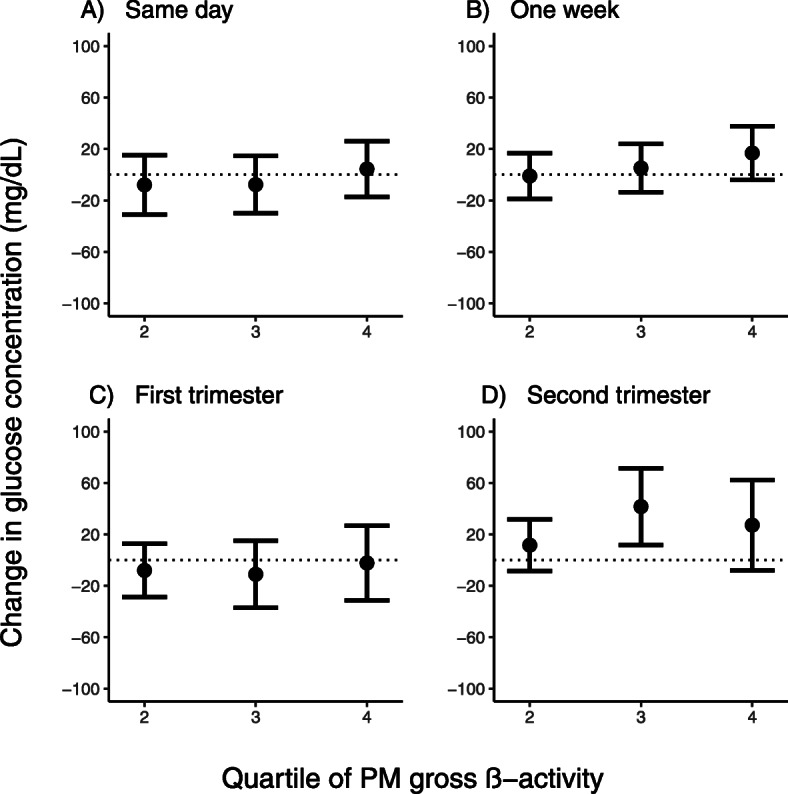
Change in glucose concentration for each quartile of PM gross β-activity concentration relative to the first quartile from the day of GCT (panel **a**), the week prior (panel **b**), the first trimester (panel **c**), and the second trimester (panel **d**). All models were adjusted for PM_2.5_, temperature, pre-pregnancy body mass index, maternal age, maternal race/ethnicity, maternal educational attainment, insurance status, median neighborhood income, median value of owner-occupied housing, and percent open space. The x-axis represents the quartile of PM gross β-activity, and the y-axis represents the change in glucose concentration in mg/dL. The error bars denote the 95% confidence intervals

Pregnancy glucose levels were broadly observed to increase non-linearly across quartiles of PM gross β-activity, with the effect of average second-trimester PM gross β-activity peaking in the third quartile (Fig. 2). Average exposure on the day of the GCT did not have a significant effect on glucose levels. Specifically, increasing quartiles of same-day exposure were associated with a change in mean glucose concentration of − 8.0 (95% CI: − 31.0, 14.9), − 6.7 (95% CI: − 28.9, 15.5), and 5.5 (95% CI: − 16.2, 27.2) mg/dL, respectively. Average PM gross β-activity exposure the week prior to the GCT and in the first trimester over exposure quartiles was non-significantly associated with a change in mean glucose concentration of − 0.7 (95% CI: − 18.8, 17.3), 5.6 (95% CI: − 13.4, 24.7), and 17.0 (95% CI: − 4.0, 38.0) mg/dL, respectively, and − 7.9 (95% CI: − 28.8, 12.9), − 11.1 (95% CI: − 37.4, 15.2), and − 2.3 (95% CI: − 31.6, 26.9) mg/dL, respectively. Consistent with the overall effect of average second-trimester exposure on glucose levels, change in glucose levels across quartiles during the second trimester also had a larger magnitude compared to the other exposure windows. Glucose levels significantly increased by 9.5 (95% CI: − 11.0, 30.0), 39.6 (95% CI: 9.7, 69.5), and 26.0 (95% CI: − 9.0, 61.1) mg/dL, respectively, with increasing second-trimester PM gross β-activity quartiles. Changes in GCT glucose level across quartiles of PM gross β-activity, with and without adjustment for PM_2.5_ were essentially the same (Supplemental Fig. 3). Simultaneous adjustment for first- and second-trimester exposures did not change the results. Similar to the results for change in the glucose concentration per IQR increase of PM gross β-activity, the results by quartile were also similar when we used multiple imputation method for insurance status (Supplemental Table 3) and in the same direction, although attenuated, when we imputed for all missing covariates (data not shown).

**Fig. 3:**
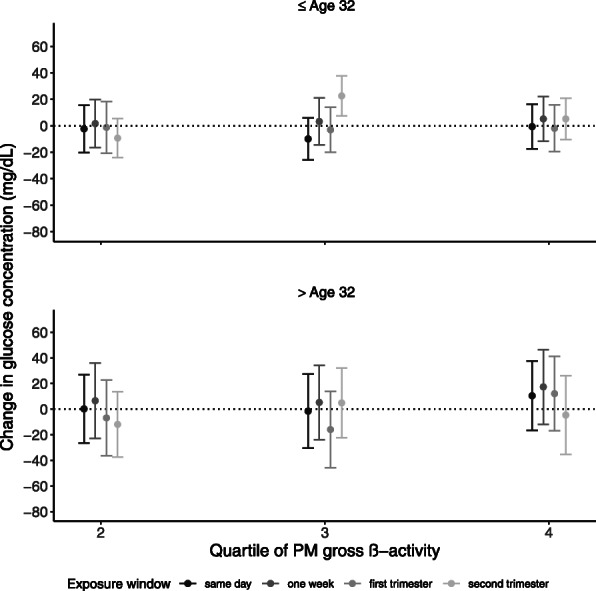
Stratified analyses by median maternal age of 32 years comparing the change in glucose concentration by quartiles of PM gross β-activity. All models were adjusted for PM_2.5_, temperature, pre-pregnancy body mass index, maternal race/ethnicity, maternal educational attainment, insurance status, median neighborhood income, median value of owner-occupied housing, and percent open space. The x-axis represents the quartile of PM gross β-activity, and the y-axis represents the change in glucose concentration in mg/dL. The error bars denote the 95% confidence intervals

In the analysis stratified by maternal age, the association between ambient PM gross β-activity and blood glucose levels was significant in younger participants (Fig. 3). Exposure to the third quartile of PM gross β-activity during the second trimester was associated with 22.6 (95% CI: 7.4, 37.8) mg/dL higher GCT glucose levels for those at or below the median maternal age of 32 (*N* = 52) and 4.9 (95% CI: − 22.4, 32.2) mg/dL higher GCT glucose levels for those above age 32 (*N* = 51). The association may also be stronger among overweight/obese (BMI ≥ 25) participants (*N* = 63) compared to underweight/normal (BMI < 25) participants (*N* = 40), although the confidence intervals are wide (Supplemental Fig. 4).

## Discussion

In our analysis of patients who received prenatal care at a single institution, exposure to higher second-trimester levels of residential PM gross β-activity was associated with higher non-fasting glucose levels measured at the time of the GCT as a part of GDM screening. Although statistically non-significant, our findings also suggest that higher exposure during the one-week window was positively associated with glucose levels. Same-day and first-trimester PM gross β-activity exposure were not associated with changes in glucose levels. Younger participants (age ≤ 32 years) had higher GCT glucose levels with increasing exposure to PM gross β-activity in the second trimester than older participants (age > 32 years). Findings from this cohort study could provide key information about a novel risk factor for an increasingly concerning pregnancy complication and its sequelae. However, our results should be interpreted with caution due to the observational nature of the study and the potential for residual confounding and other biases.

Although each exposure window of PM gross β-activity that we examined followed the same overall pattern of higher pregnancy glucose levels with higher exposure, average exposure to PM gross β-activity over the second trimester was observed to have a larger magnitude of increase in glucose concentration than smaller exposure windows. This finding suggests that the second trimester is a sensitive window of exposure as it relates to glucose tolerance in pregnancy. Indeed, the second trimester is a period of increased insulin resistance thought to be due at least in part to the higher levels of several hormones resulting from the growing placenta, including circulating placental lactogen, progesterone, prolactin, placental growth hormone, and cortisol. Tumor necrosis factor α and leptin have also been implicated as contributors to insulin resistance and hyperglycemia in pregnancy [39, 40]. Further, it is possible that any disruption of normal glucose metabolism may require a cumulative rather than acute exposure window, which may explain why stronger associations were seen for the average second-trimester window compared to the week prior to the GCT. The finding that second trimester is a sensitive window of exposure for higher pregnancy glucose levels aligns with findings from previous studies evaluating the association between PM_2.5_ and GDM. In fact, a recent systematic review of cohort studies found that only second-trimester exposure affects glucose homeostasis [10]. As such, this present study may further support the possibility that the radiometric component of PM could not only impact GDM, but also, more subtly, pregnancy glucose levels that do not meet the clinical threshold for GDM.

When comparing the highest with the lowest quartile of PM gross β-activity exposure, we found a pattern for elevated GCT glucose levels for the same-day, one-week, and second-trimester exposure windows. High glucose concentration during pregnancy could be a precursor to GDM, a well-established risk factor for health complications during pregnancy and later in life for both mother and child [41]. In a pregnant population, Papatheodorou et al. [20] found that the highest quartile of PM gross β-activity exposure during the second trimester was associated with higher odds of GDM compared to the lowest quartile (*OR* = 1.08, 95% CI: 1.04, 1.13), the same exposure window in which the present study also found a statistically significant association for. Previous work on the effect of radioactivity on glucose level has largely been conducted in non-pregnant populations, especially cancer patients who are exposed to low doses of external radiation as part of cancer treatment. Although exposure to PM gross β-activity is distinguished from radiotherapy by being a source of internal radiation, their effects and mechanisms may overlap. One study demonstrated an increase in average blood glucose concentration by 14.7 mg/dL (12.8%) during treatment compared to prior to radiotherapy among glioblastoma multiforme patients [42]. The investigators also demonstrated a positive dose-response between radiotherapy and blood glucose. In another study among patients without diabetes who underwent chemoradiation for head or neck cancer, average glucose levels increased for the entire 10 weeks of treatment, with glucose level changes as high as 15.45 mg/dL in week 8 compared to before treatment [43]. The Childhood Cancer Survivor Study, which included 8599 long-term childhood cancer survivors, showed them to have 1.8 times the odds of having diabetes mellitus compared to siblings later in life [44]. Nylander et al. suggests that epigenetic mechanisms may play a role in the development of insulin resistance from exposure to ionizing radiation [45]. While high doses of radiation may contribute to the development of diabetes, the risk of inhaled low-dose environmental radiation is not characterized well. Without more statistical power, we were unable to shed light on the effects of low dose exposure. Because there is more data on Rn, an important source of exposure to background radiation, future studies should investigate the effect of Rn gas on glucose levels to better understand the impact of low-dose radiation exposure.

The association between PM gross β-activity and pregnancy glucose levels was stronger in participants ≤32 years old compared to those who were > 32 years old. Because we were severally underpowered to formally assess for effect measure modification, lack of an observed associate in either age strata does not necessarily mean that there is none. Furthermore, what our findings suggest is consistent with what was previously observed between second-trimester PM_2.5_ and GDM. Fleisch et al. [24] found that the odds of GDM were highest among younger mothers and decreased with maternal age. Because maternal age is a strong risk factor for GDM, we may expect younger mothers to be more affected by environmental exposures, including air pollution, than older mothers, who may be more likely to develop GDM regardless of other exposures. Children and young adults’ respiratory health has been understood to be more heavily impacted by PM_2.5_ exposure compared to older adults due to physiological development and lifestyle [46].

Our study has several limitations. We had limited power due to the small sample size given that we used only a subset of the ongoing ERGO cohort that had available information on both PM gross β-activity and pregnancy glucose levels from the GCT. Because individual characteristics in this subset were similar to those in the overall ERGO cohort to date, it is unlikely that our sample suffered from selection bias due to this restriction. While participants in ERGO were screened for GDM using the 2-step method, diabetic patients or patients that providers suspect may have diabetes are often given alternative GDM tests in practice [47], and our findings may not be generalizable to these cases. Although we used beta activity from only two monitoring locations, Blomberg AJ et al. [27] found high correlations between state and regional average daily particle beta activity concentrations. This demonstrates that capturing variability within a region is more important than covering a large geographic area, and we had PM gross β-activity values for several days per week. Consequently, assigning exposures based on zip code of residence at the time of the GCT is not expected to introduce significant misclassification of exposure. Nevertheless, the exposure misclassification is likely to be non-differential, resulting in bias towards the null. That is, each participant’s assigned PM gross β-activity based on residential zip-code, whether higher or lower compared to the participant’s actual exposure level, is unlikely to be related to their observed glucose level and can lead to an underestimation of the true association. Future studies should use a larger sample size and more granular spatial resolution to confirm our findings. Since the present study only considers residential exposure, future works may also want to consider time-varying patterns and the contribution of ambient occupational exposures. Although our study gives insight about exposure to radiation through inhalation, which constitutes the majority of natural background radiation exposure [18], we lack information about radiation from other routes of exposure that may influence glucose levels, which can be taken into consideration in subsequent studies. Although there is large uncertainty in the change in glucose concentration estimates, the findings suggest that future work is needed to determine whether average second-trimester PM gross β-activity can be a modifiable risk factor, possibly through reducing exposure to air pollution, for GDM or other pregnancy complications that are linked to high glucose levels during pregnancy.

This study has several strengths. First, to our knowledge, this study is the first to evaluate the relationship between ambient PM gross β-activity and blood glucose levels in a pregnant population, although prior work on GDM as a health endpoint in a sense is similar. Second, we used clinical measures of pregnancy glucose levels to better understand whether PM gross β-activity can alter glucose levels during the increasingly insulin resistant state of pregnancy. Third, we evaluated multiple exposure windows, which allowed for assessment of acute and cumulative exposures, finding that second trimester was a particularly sensitive window. Fourth, we assessed the association by maternal age, which showed that younger participants may be more susceptible to this novel environmental factor as it relates to pregnancy glucose levels.

## Conclusions

Exposure to higher levels of PM gross β-activity during certain periods of pregnancy were associated with higher pregnancy glucose levels in our study. This finding could have clinical implications, especially for young mothers. Due to the observational nature of the present study, we cannot claim causal conclusions but only provide evidence of a statistically significant association. Future work is needed to determine whether reduction in exposure to this potentially modifiable environmental risk factor, particularly in the second trimester of pregnancy, could reduce the risk of developing GDM and other related adverse health outcomes linked to this condition.

## Supplementary Information

**Additional file 1.**

## Data Availability

The data that support the findings of this study are available on request from the corresponding author SP. The data are not publicly available due to them containing information that could compromise research participant privacy/consent.

## References

[1] Hunt KJ, Schuller KL (2007). The increasing prevalence of diabetes in pregnancy. Obstet Gynecol Clin N Am.

[2] Xiao RS, Simas TAM, Person SD, Goldberg RJ, Waring ME. Diet quality and history of gestational diabetes mellitus among childbearing women, United States, 2007–2010. Prev Chronic Dis. 201526;12:1–9. 10.5888/pcd12.140360 PMC4344352 25719215

[3] Deputy NP. Prevalence and changes in preexisting diabetes and gestational diabetes among women who had a live birth — United States, 2012–2016. MMWR Morb Mortal Wkly Rep. 2018;67:1201–7. 10.15585/mmwr.mm6743a2 PMC6319799 30383743

[4] Kim C, Newton KM, Knopp RH (2002). Gestational diabetes and the incidence of type 2 diabetes: a systematic review. Diab Care.

[5] CDC. Gestational Diabetes and Pregnancy | CDC [Internet]. Centers for Disease Control and Prevention. 2020. Available from: https://www.cdc.gov/pregnancy/diabetes-gestational.html.

[6] Casagrande SS, Linder B, Cowie CC (2018). Prevalence of gestational diabetes and subsequent type 2 diabetes among U.S. women. Diab Res Clin Pract.

[7] Vounzoulaki E, Khunti K, Abner SC, Tan BK, Davies MJ, Gillies CL (2020). Progression to type 2 diabetes in women with a known history of gestational diabetes: systematic review and meta-analysis. BMJ..

[8] The American College of Obstetricians and Gynecologists. ACOG Practice Bulletin No. 201: Pregestational Diabetes Mellitus. Obstet Gynecol. 2018;132(6):e228–48. 10.1097/AOG.0000000000002960 30461693

[9] Carr DB, Newton KM, Utzschneider KM, Tong J, Gerchman F, Kahn SE, Heckbert SR (2008). Modestly elevated glucose levels during pregnancy are associated with a higher risk of future diabetes among women without gestational diabetes mellitus. Diab Care.

[10] Tang X, Zhou J-B, Luo F, Han Y, Heianza Y, Cardoso MA, Qi L (2020). Air pollution and gestational diabetes mellitus: evidence from cohort studies. BMJ Open Diabetes Res Care.

[11] Schlesinger RB (2007). The health impact of common inorganic components of fine particulate matter (PM2.5) in ambient air: a critical review. Inhal Toxicol.

[12] Li W, Nyhan MM, Wilker EH, Vieira CLZ, Lin H, Schwartz JD, Gold DR, Coull BA, Aba AM, Benjamin EJ, Vasan RS, Koutrakis P, Mittleman MA (2018). Recent exposure to particle radioactivity and biomarkers of oxidative stress and inflammation: the Framingham heart study. Environ Int.

[13] Porstendoerfer J (2001). Physical parameters and dose factors of the radon and thoron decay products. Nucl Technol Publ.

[14] Mostafa AMA, Tamaki K, Moriizumi J, Yamazawa H, Iida T (2011). The weather dependence of particle size distribution of indoor radioactive aerosol associated with radon decay products. Radiat Prot Dosim.

[15] Mostafa AMA, Yamazawa H, Uosif MAM, Moriizumi J (2015). Seasonal behavior of radon decay products in indoor air and resulting radiation dose to human respiratory tract. J Radiat Res Appl Sci.

[16] Mohamed A, Ahmed AA, Ali AE, Yuness M. Attached and unattached activity size distribution of short-lived radon progeny (214Pb) and evaluation of deposition fraction. 2008;9.

[17] Keith S, Doyle JR, Harper C, Mumtaz M, Tarrago O, Wohlers DW, et al. Toxicological Profile for Radon [Internet]. Toxicological Profile for Radon. Agency for Toxic Substances and Disease Registry (US); 2012. p. 15–106. Available from: https://www.ncbi.nlm.nih.gov/books/NBK158780/. 24049860

[18] CDC. Radiation from the Earth (Terrestrial Radiation) [Internet]. Centers for Disease Control and Prevention. 2016. Available from: https://www.cdc.gov/nceh/radiation/terrestrial.html

[19] Hernández F, Hernández-Armas J, Catalán A, Fernández-Aldecoa JC, Karlsson L (2005). Gross alpha, gross beta activities and gamma emitting radionuclides composition of airborne particulate samples in an oceanic island. Atmos Environ.

[20] Papatheodorou S, Gold DR, Blomberg AJ, Hacker M, Wylie BJ, Requia WJ, Oken E, Fleisch AF, Schwartz JD, Koutrakis P (2020). Ambient particle radioactivity and gestational diabetes: a cohort study of more than 1 million pregnant women in Massachusetts, USA. Sci Total Environ.

[21] Sonagra AD, Biradar SM, K D, Murthy DSJ (2014). Normal pregnancy- a state of insulin resistance. JCDR.

[22] Environmental Reproductive and Glucose Outcomes Study (ERGO) [Internet]. Available from: https://ergo.sph.harvard.edu/home.

[23] US EPA O. RadNet [Internet]. US EPA. 2014. Available from: https://www.epa.gov/radnet.

[24] Moriizumi J, Yamada S, Xu Y, Matsuki S, Hirao S, Yamazawa H (2014). Indoor/outdoor radon decay products associated aerosol particle-size distributions and their relation to total number concentrations. Radiat Prot Dosim.

[25] US EPA. Radiological Laboratory Sample Analysis Guide for Incidents of National Significance – Radionuclides in Air. 2009;101.

[26] Breiman L (2001). Random forests. Mach Learn.

[27] Blomberg AJ, Li L, Schwartz JD, Coull BA, Koutrakis P (2020). Exposure to particle Beta radiation in greater Massachusetts and factors influencing its spatial and temporal variability. Environ Sci Technol.

[28] NICHD. About Pregnancy [Internet]. https://www.nichd.nih.gov/. 2017. Available from: https://www.nichd.nih.gov/health/topics/pregnancy/conditioninfo.

[29] Carpenter MW, Coustan DR (1982). Criteria for screening tests for gestational diabetes. Am J Obstet Gynecol.

[30] American Diabetes Association (2013). Diagnosis and classification of diabetes mellitus. Diab Care.

[31] Mesinger F, DiMego G, Kalnay E, Mitchell K, Shafran PC, Ebisuzaki W, et al. North American Regional Reanalysis. Bull Am Meteorol Soc. 2006;87(3):343–60.

[32] Farah N, McGoldrick A, Fattah C, O’Connor N, Kennelly MM, Turner MJ (2012). Body mass index (BMI) and glucose intolerance during pregnancy in white European women. J Reprod Infertil.

[33] McFarland KF, Case CA (1985). The relationship of maternal age on gestational diabetes. Diab Care.

[34] MassGIS. Massachusetts Document Repository [Internet]. 2020. Available from: https://docs.digital.mass.gov/dataset/massgis-data-layers?_ga=2.119059215.727478168.1588279152-148147306.1587403954#cen.

[35] Fleisch AF, Kloog I, Luttmann-Gibson H, Gold DR, Oken E, Schwartz JD. Air pollution exposure and gestational diabetes mellitus among pregnant women in Massachusetts: a cohort study. Environ Health [Internet]. 2016;15(40). Available from: https://dash.harvard.edu/handle/1/25658529. 10.1186/s12940-016-0121-4 PMC4765142 26911579

[36] Choe S-A, Eliot MN, Savitz DA, Wellenius GA (2019). Ambient air pollution during pregnancy and risk of gestational diabetes in New York City. Environ Res.

[37] CDC. All About Adult BMI [Internet]. Centers for Disease Control and Prevention. 2020. Available from: https://www.cdc.gov/healthyweight/assessing/bmi/adult_bmi/index.html.

[38] Donovan L, Hartling L, Muise M, Guthrie A, Vandermeer B, Dryden DM (2013). Screening tests for gestational diabetes: a systematic review for the U.S. preventive services task force. Ann Intern Med.

[39] Barbour LA, McCurdy CE, Hernandez TL, Kirwan JP, Catalano PM, Friedman JE (2007). Cellular mechanisms for insulin resistance in normal pregnancy and gestational diabetes. Diab Care.

[40] Lowe LP, Metzger BE, Lowe WL, Dyer AR, McDade TW, McIntyre HD (2010). Inflammatory mediators and glucose in pregnancy: results from a subset of the hyperglycemia and adverse pregnancy outcome (HAPO) study. J Clin Endocrinol Metab.

[41] Kc K, Shakya S, Zhang H (2015). Gestational diabetes mellitus and Macrosomia: a literature review. Ann Nutr Metab.

[42] Duma MN, Oszfolk NI, Boeckh-Behrens T, Oechsner M, Zimmer C, Meyer B, Pfluger PT, Combs SE (2019). Positive correlation between blood glucose and radiotherapy doses to the central gustatory system in Glioblastoma Multiforme patients. Radiat Oncol.

[43] Nguyen NP, Vos P, Vinh-Hung V, Borok TL, Dutta S, Karlsson U, Lee H, Martinez T, Jo BH, Nguyen LM, Nguyen N, Sallah S (2009). Altered glucose metabolism during Chemoradiation for head and neck Cancer. Anticancer Res.

[44] Meacham LR, Sklar CA, Li S, Liu Q, Gimpel N, Yasui Y, Whitton JA, Stovall M, Robison LL, Oeffinger KC (2009). Diabetes mellitus in long-term survivors of childhood Cancer: increased risk associated with radiation therapy a report for the childhood Cancer survivor study (CCSS). Arch Intern Med.

[45] Nylander V, Ingerslev LR, Andersen E, Fabre O, Garde C, Rasmussen M, Citirikkaya K, Bæk J, Christensen GL, Aznar M, Specht L, Simar D, Barrès R (2016). Ionizing radiation potentiates high-fat diet–induced insulin resistance and reprograms skeletal muscle and adipose progenitor cells. Diabetes..

[46] Buka I, Koranteng S, Osornio-Vargas AR (2006). The effects of air pollution on the health of children. Paediatr Child Health.

[47] Nicklas JM, Zera CA, Lui J, Seely EW. Patterns of gestational diabetes diagnosis inside and outside of clinical guidelines. BMC Pregnancy Childbirth [Internet]. 2017;17(11). Available from: https://www.ncbi.nlm.nih.gov/pmc/articles/PMC5219746/. 10.1186/s12884-016-1191-6 PMC5219746 28061829

