# Supplementary material for: Ambient PM gross β-activity and glucose levels during pregnancy
Source: Environ Health. 2021 Jun 14;20:70. doi: 10.1186/s12940-021-00744-9 (PMC8204493; doi:10.1186/s12940-021-00744-9)
Supplement: Supplementary file 1 — Additional file 1. [file 12940_2021_744_MOESM1_ESM.docx]

Supplementary Material

Supplemental Table 1. Compares the socio-demographic characteristics of the ERGO subset included in the present study (103 women) to those of all ERGO participants.

|  | **Present study* (N=103)** | **Overall ERGO (N=117)** |
| --- | --- | --- |
| **Age, years** | 32.7 (4.6) | 32.9 (4.5) |
| **Highest educational attainment** | | |
| <HS | 0 (0.0%) | 1 (0.9%) |
| HS/College | 48 (46.6%) | 58 (49.6%) |
| Graduate school | 55 (53.4%) | 58 (49.6%) |
| **Race/ethnicity** | | |
| White | 68 (66.0%) | 15 (60.0%) |
| Non-White | 35 (34.0%) | 10 (40.0%) |
| **BMI** | 24.9 (4.7) | 24.5 (3.4) |
| **Prenatal insurance type** | | |
| Private | 88 (85.4%) | 98 (84.5%) |
| Other | 15 (14.6%) | 18 (15.5%) |

Values of continuous variables were reported as mean (SD), while values of categorical variables were reported as n (%).

*The cohort used in the present study is not a subset of the overall ERGO cohort shown in this table. Summary statistics of the overall ERGO cohort were generated prior to the present study.

Supplemental Table 2. Correlation matrix of first and second trimester PM gross β-activity and PM_2.5_.

|  | First trimester  PM gross β-activity | Second trimester  PM gross β-activity | First trimester  PM_2.5_ | Second trimester  PM_2.5_ |
| --- | --- | --- | --- | --- |
| First trimester  PM gross β-activity | 1 | - | - | - |
| Second trimester  PM gross β-activity | -0.53* | 1 | - | - |
| First trimester  PM_2.5_ | 0.18 | -0.30* | 1 | - |
| Second trimester  PM_2.5_ | -0.30* | 0.11 | -0.12 | 1 |

*p<0.05

Supplemental Table 3. Changes in glucose concentration overall (per IQR increase) and by quartile of PM gross β-activity after imputing missing values of insurance status.

|  | Difference in glucose level (95% CI), mg/dL | | | |
| --- | --- | --- | --- | --- |
| Exposure window | Overall^a^ | Quartile 2^b^ | Quartile 3^b^ | Quartile 4^b^ |
| Same-day | 3.8 (-6.9, 14.4) | -11.5 (-31.8, 8.7) | -8.9 (-28.2, 11.4) | 2.17 (-16.5, 22.4) |
| One-week | 6.5 (-1.6, 14.6) | -0.6 (-17.4, 16.1) | 5.5 (-12.2, 22.2) | 17.6 (-2.3, 34.3) |
| First trimester | 6.7 (-8.4, 21.8) | -5.7 (-24.4, 13.1) | -9.0 (-30.6, 9.8) | -3.2 (-28.9, 15.6) |
| Second trimester | 16.3 (0.2, 32.4) | 10.8 (-8.5, 30.2) | 38.0 (9.0, 57.3) | 20.8 (-13.4, 40.2) |

^a^per interquartile range of PM gross β-activity

^b^compared to quartile 1

Supplemental Fig. 1


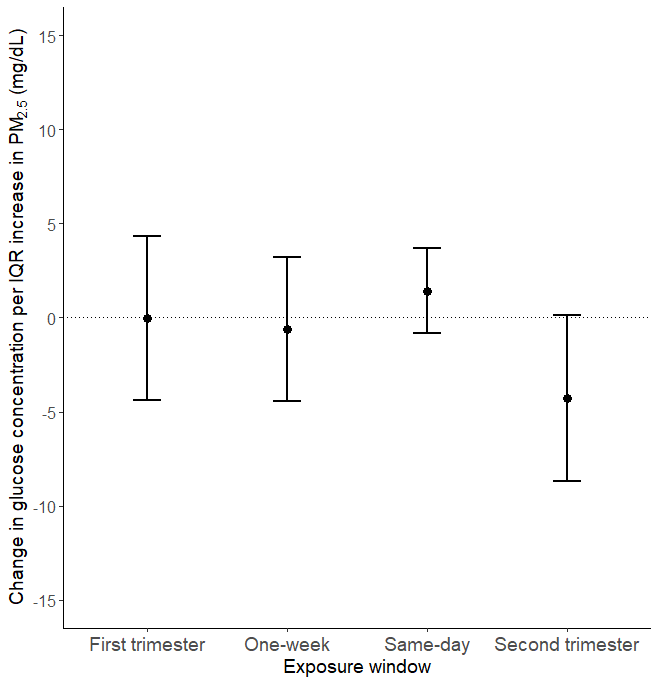


Changes in glucose concentration per IQR increase in PM_2.5_ for each exposure window: the day of GCT examination, the week prior, the first trimester, and the second trimester. All models were adjusted for temperature, pre-pregnancy body mass index, maternal race/ethnicity, maternal educational attainment, insurance status, median neighborhood income, median value of owner-occupied housing, and percent open space. The x-axis represents the exposure window, and the y-axis represents the change in glucose concentration in mg/dL per interquartile range (IQR) increase in PM_2.5_. The error bares denote the 95% confidence intervals.

Supplemental Fig. 2


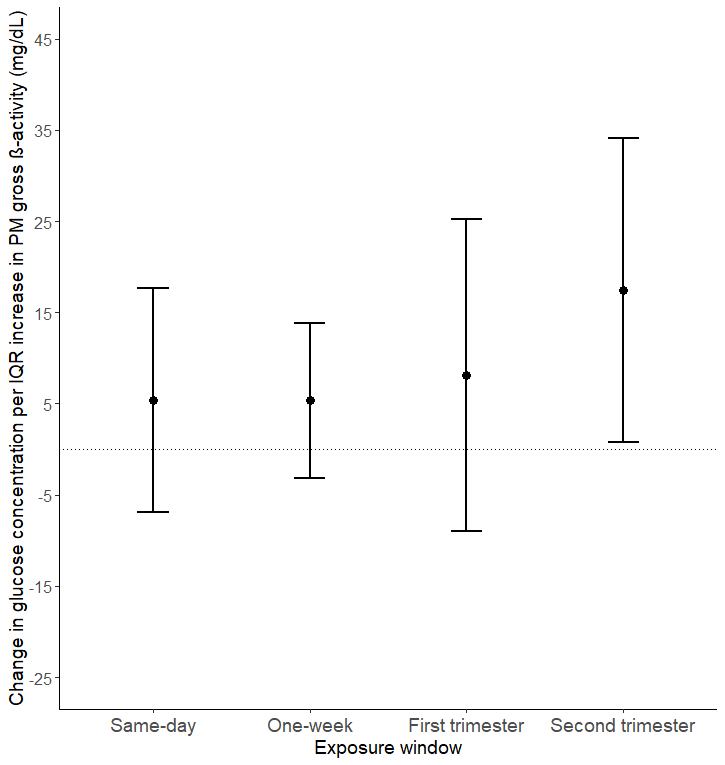


Changes in glucose concentration per IQR increase in PM gross β-activity for each exposure window: the day of GCT examination, the week prior, the first trimester, and the second trimester. All models were adjusted for temperature, pre-pregnancy body mass index, maternal race/ethnicity, maternal educational attainment, insurance status, median neighborhood income, median value of owner-occupied housing, and percent open space. The x-axis represents the exposure window, and the y-axis represents the change in glucose concentration in mg/dL per interquartile range (IQR) increase in PM gross β-activity. The error bares denote the 95% confidence intervals.

Supplemental Fig. 3


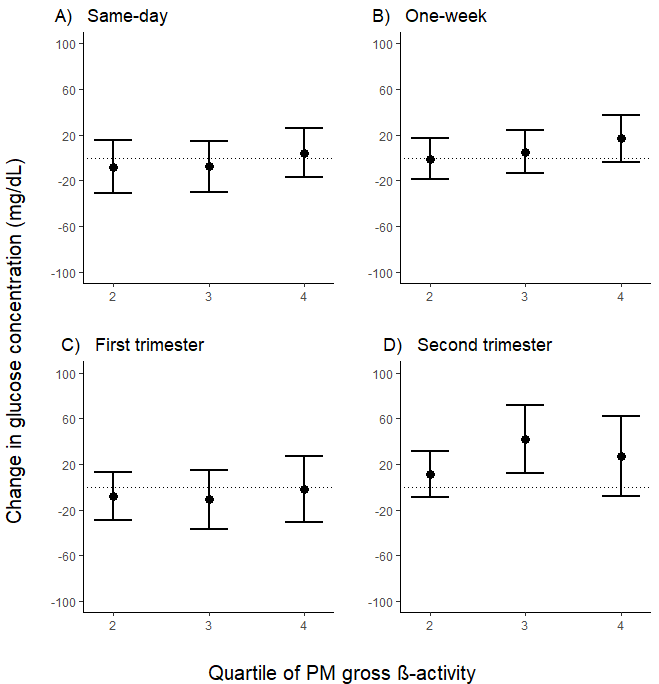


Change in glucose concentration for each quartile of PM gross β-activity concentration relative to the first quartile from the day of GCT examination (panel A), the week prior (panel B), the first trimester (panel C), and the second trimester (panel D). All models were adjusted for temperature, pre-pregnancy body mass index, maternal age, maternal race/ethnicity, maternal educational attainment, insurance status, median neighborhood income, median value of owner-occupied housing, and percent open space. The x-axis represents the quartile of PM gross β-activity, and the y-axis represents the change in glucose concentration in mg/dL. The error bares denote the 95% confidence intervals.

Supplemental Fig. 4


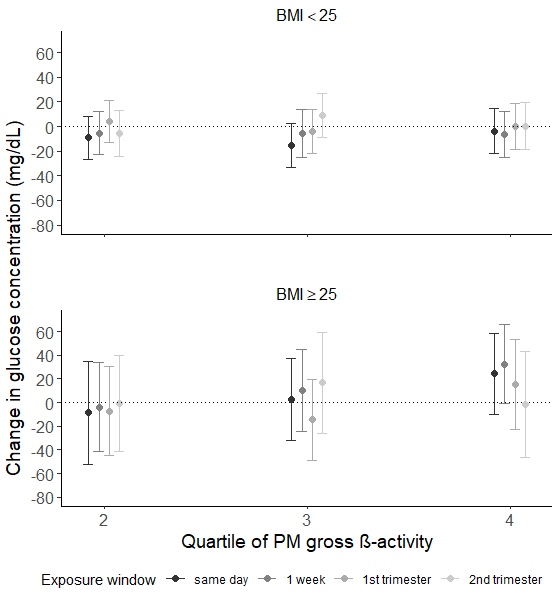


Stratified analyses by BMI of 25 comparing the change in glucose concentration by quartiles of PM gross β-activity. All models were adjusted for PM_2.5_, temperature, pre-pregnancy body mass index, maternal race/ethnicity, maternal educational attainment, insurance status, median neighborhood income, median value of owner-occupied housing, and percent open space. The x-axis represents the quartile of PM gross β-activity, and the y-axis represents the change in glucose concentration in mg/dL. The error bars denote the 95% confidence intervals.
